# Low DAPK1 expression correlates with poor prognosis and sunitinib resistance in clear cell renal cell carcinoma

**DOI:** 10.18632/aging.103638

**Published:** 2020-11-16

**Authors:** Zhengshuai Song, Zhongyuan Li, Weiwei Han, Chenxi Zhu, Ning Lou, Xuechao Li, Gang Luo, Song Peng, Guohao Li, Ye Zhao, Yonglian Guo

**Affiliations:** 1 Department of Urology, The Central Hospital of Wuhan, Tongji Medical College, Huazhong University of Science and Technology, Wuhan, China; 2 Cancer Center, Union Hospital, Tongji Medical College, Huazhong University of Science and Technology, Wuhan, China

**Keywords:** ccRCC, DAPK1, sunitinib resistance, biomarker, ER stress

## Abstract

We investigated the prognostic significance of Death-Associated Protein Kinase 1 (DAPK1) and its role in sunitinib resistance in clear cell renal cell carcinoma (ccRCC). DAPK1 mRNA levels were significantly lower in tumor tissues than normal kidney tissues in TCGA-KIRC dataset (n=428). Both overall survival and disease-free survival were significantly shorter in ccRCC patients with low DAPK1 expression than those with high DAPK1 expression. Receiver operating characteristic curve analysis showed that low DAPK1 expression correlated with poor prognosis in ccRCC patients. Multivariate analysis confirmed that DAPK1 expression was an independent prognostic indicator in ccRCC. Gene set enrichment analysis showed that low DAPK1 expression correlates with upregulation of pathways related to metastasis, drug resistance, hypoxia and invasiveness in ccRCC patients. Sunitinib-resistant ccRCC cells show significantly lower DAPK1 mRNA and protein levels than sunitinib-sensitive ccRCC cells. DAPK1 overexpression enhances apoptosis in sunitinib-resistant ccRCC cells via the ATF6-dependent ER stress pathway. Xenograft tumors derived from DAPK1-overxpressing ccRCC cells were significantly smaller than the controls in nude mice. Our finding demonstrates that low DAPK1 expression is an independent prognostic indicator that correlates with ccRCC progression and sunitinib resistance.

## INTRODUCTION

In 2018, renal cell cancer (RCC), which is the most common and lethal urological malignancy, accounted for 403,262 new cases and 175,098 deaths worldwide [1]. In 2019, RCC accounted for nearly 73,820 new cases and 14,770 deaths in the United States [2]. The incidence of RCC has risen gradually over the last two decades, and nearly 30% of ccRCC patients are diagnosed with metastatic cancer [3, 4]. Clear cell renal cell carcinoma (ccRCC) accounts for the majority of RCC-related cases and deaths [5]. There have been significant improvements in RCC treatments in the last decade, including tyrosine kinase inhibitors (TKIs) that the availability of specific molecular target drugs, such as sunitinib, but, the prognosis of advanced ccRCC patients remains very poor [6]. In addition to late diagnosis, the survival rates of advanced ccRCC patients are poor because majority of patients develop drug resistance after a brief period of remission [7]. Therefore, new and more effective biomarkers that can predict metastasis or drug resistance are urgently required to improve survival rates of ccRCC patients with metastatic cancer and drug resistance.

*DAPK1* gene is conserved in the evolution of many invertebrates, chordates and mammals, and belongs to a family of serine-threonine kinases that also includes *DAPK2, DAPK3, DRAK1*, and *DRAK2* [8]. DAPK1 is a calcium/calmodulin–regulated (CaM-regulated) protein kinase that induces apoptosis in response to various stimuli such as IFN-γ, TNF-α, and TGF-β [9–11]. DAPK1 is a pivotal component in the endoplasmic reticulum (ER) stress-induced cell death pathway [12]. It mediates ER stress signaling and activates autophagy as well as caspase-dependent cell death in response to multiple intracellular and extracellular stimuli [13, 14]. Previous studies also show that DAPK1 is a tumor suppressor gene that inhibits tumor growth and metastasis by promoting apoptosis and autophagy [15]. The functional role of DAPK1 in renal cancer growth, progression and drug resistance has not been reported. Therefore, in this study, we investigated the prognostic significance of DAPK1 and its role in the sunitinib resistance mechanisms in ccRCC patients.

## RESULTS

### Low DAPK1 expression is associated with tumor progression and metastasis in ccRCC

**Figure 1:**
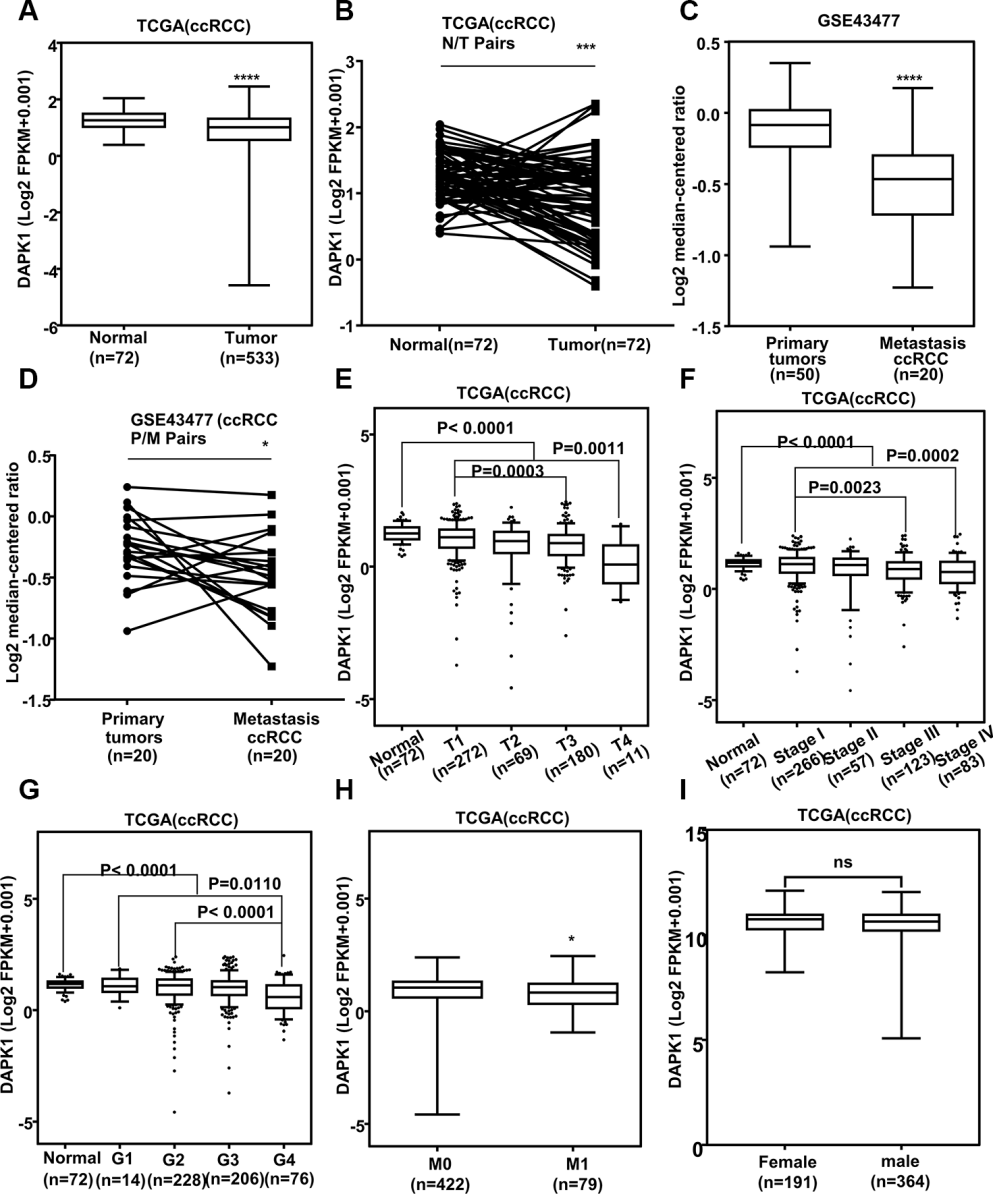
**Low DAPK1 expression correlates with tumor progression and metastasis in ccRCC patients.** (**A**) DAPK1 mRNA expression in ccRCC (n=531) and adjacent normal kidney tissues (n=72) from the The Cancer Genome Atlas-Kidney Renal clear cell Carcinoma (TCGA**-**KIRC) database. (**B**) DAPK1 mRNA expression in paired ccRCC and para**-**cancerous kidney tissues (n=72) from the TCGA**-**KIRC database. (**C**) DAPK1 mRNA expression in primary (n=50) and metastatic ccRCC (n=20) tumors from the GSE43477 dataset. (**D**) DAPK1 mRNA expression in paired primary and metastatic ccRCC tumors (n=20) from the GSE43477 database. (**E**–**I**) Correlation between DAPK1 mRNA expression and clinicopathological parameters, including (**E**) T stage (T1-T4), (**F**) pathologic stage (I-IV), (**G**) tumor grade (G1-G4), (**H**) distant metastases, and (**I**) gender (male or female). Note: The data are shown as means ± SEM; ***p<0.001, **p<0.01, *p<0.05.

The relationship between DAPK1 mRNA levels and clinicopathological characteristics such as age, gender, tumor size (T), node (N) and metastasis (M), tumor grade, and tumor stage of ccRCC patients (n=428) from the TCGA-KIRC database are shown in Table 1. DAPK1 mRNA levels were significantly lower in ccRCC tissues compared to the adjacent normal kidney tissues in the ccRCC patient tissues (n=428) from the TCGA-KIRC database (Figure 1A). Furthermore, analysis of 72 paired ccRCC and adjacent normal kidney tissues in TCGA-KIRC database showed similar results (Figure 1B). DAPK1 mRNA expression was significantly decreased in the metastatic ccRCC tissues compared to the primary ccRCC tumor tissues in the GSE43477 ccRCC dataset (Figure 1C, 1D). Moreover, low DAPK1 expression was associated with advanced tumor characteristics such as T stage (Figure 1E), pathological TNM stage (Figure 1F), tumor grade (Figure 1G) and distant metastasis (Figure 1H), but there was no correlation between DAPK1 expression and gender (Figure 1I). These data suggest that DAPK1 is downregulated in ccRCC tissues and low DAPK1 expression is closely related with ccRCC progression and metastasis.

**Table 1:** Correlation between DAPK1 mRNA expression and clinicopathological parameters of ccRCC patients from TGCA-KIRC database.

**Clinical parameters**			**DAPK1 mRNA expression**		
		**Number**	**Low (n=214)**	**High (n=214)**	**P value**
**Age (years)**	< 60	218	102	116	0.334
	≥ 60	210	109	101	
**Gender**	Female	145	65	80	0.220
	Male	283	146	137	
**T stage**	T1 or T2	286	128	158	0.01
	T3 or T4	142	83	59	
**N stage**	N0 or NX	415	206	209	0.576
	N1	13	5	8	
**M stage**	M0 or MX	375	179	196	0.106
	M1	53	32	21	
**G grade**	G1 or G2	212	95	117	0.067
	G3 or G4	216	116	100	
**TNM stage**	I + II	273	121	152	0.007
	III + IV	155	90	65	

### DAPK1 is a prognostic biomarker in ccRCC patients

**Figure 2:**
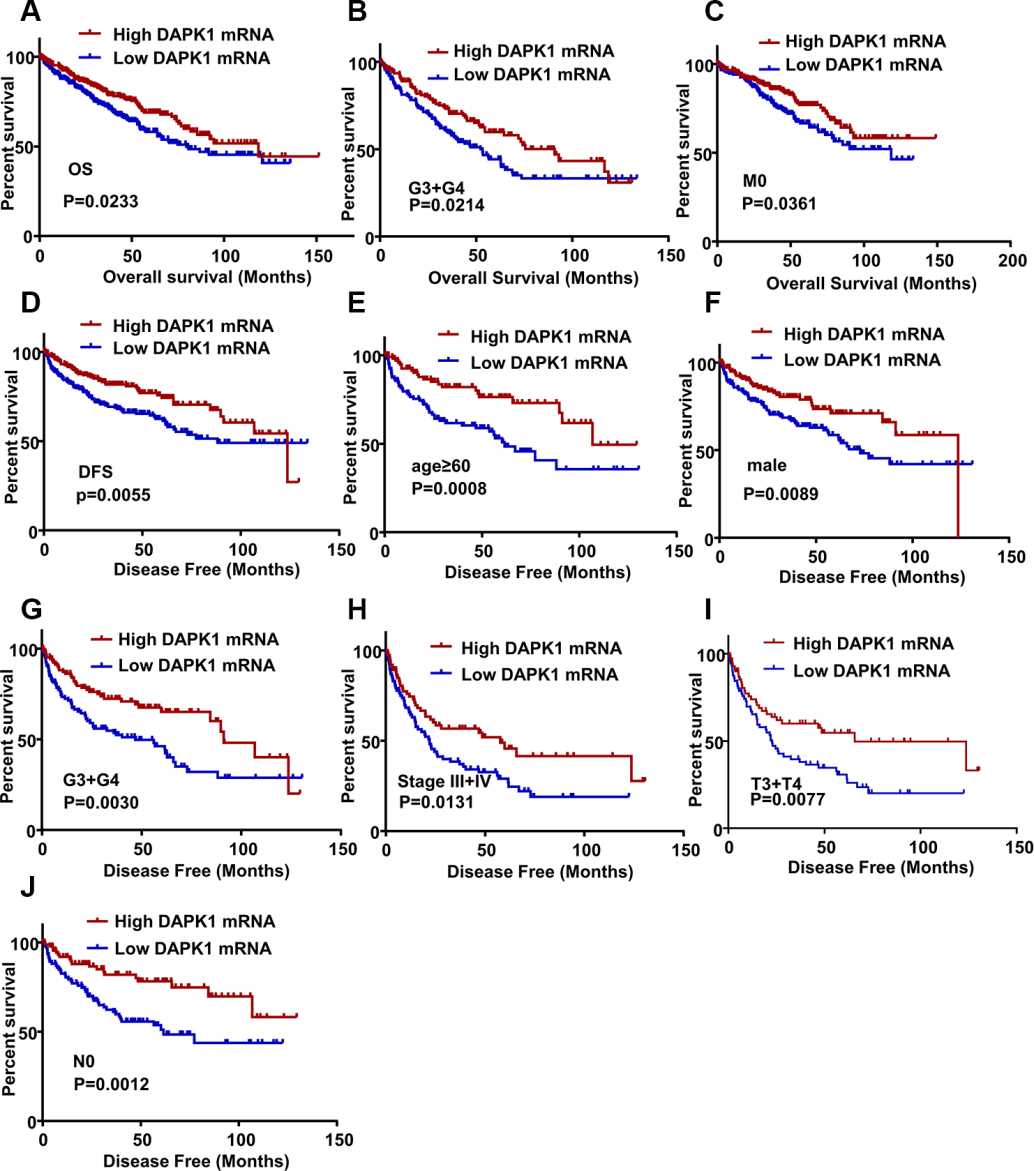
**DAPK1 expression correlates with OS and DFS of ccRCC patients.** (**A**) Kaplan-Meier survival curve analysis shows the overall survival (OS) rates of ccRCC patients with low and high DAPK1 expression. (**B**, **C**) Kaplan-Meier survival curve analysis shows OS of ccRCC patients belonging to (**B**) G3 + G4 tumor grades and (**C**) M0 stage tumors based on low or high DAPK1 expression. (**D**) Kaplan-Meier survival curve analysis shows disease-free survival (DFS) rates of ccRCC patients with low or high DAPK1 expression. (**E**–**J**) Kaplan-Meier survival curve analysis shows DFS rates based on low or high DAPK1 expression in different patient subgroups, including (**E**) age ≥ 60 y, (**F**) male, (**G**) G3 + G4 stage (**H**) pathologic stages III + IV, (**I**) T3 + T4 stage, and (**J**) N0 stage.

Next, in TCGA-KIRC database, we performed Kaplan-Meier survival curve analysis to determine the association between DAPK1 expression and overall survival (OS) as well as disease-free survival (DFS) of ccRCC patients. The ccRCC patients were categorized into low- and high-DAPK1 expression groups based on the median DAPK1 mRNA expression. The ccRCC patients with low DAPK1 expression showed significantly shorter OS (N=537, P=0.0233) and DFS (N=434, P=0.0055) compared to patients with high DAPK1 expression (Figure 2A, 2D). Subgroup analysis showed that OS was significantly shorter in ccRCC patients with low DAPK1 expression compared to those with higher DAPK1 expression belonging to G3 + G4 (Figure 2B, N=285, P=0.0214) and M0 stages (Figure 2C, N=425, P=0.0361). However, it had no significant relationship between the DAPK1 mRNA expression and OS of ccRCC with N stage and TNM stage (data not shown). Moreover, DFS was significantly shorter in ccRCC patients with lower DAPK1 expression compared to those with higher DAPK1 expression in the age ≥ 60 y (Figure 2E, N=214, P=0.0008), males (Figure 2F, N=286, P=0.0089), G3 + G4 (Figure 2G, N=217, P=0.0030), stages III + IV (Figure 2H, N=154, P=0.0131), stages T3 + T4 (Figure 2I, N=143, P=0.0077), and stage N0 (Figure 2J, N=192, P=0.0012) subgroups. DAPK1 was significantly lower in dead ccRCC patients compared to those alive in TCGA-KIRC dataset. (Supplementary Figure 1A). But, it had no significant relationship between the DAPK1 mRNA expression and DFS of ccRCC with N1 stage, M stage and female (data not shown). This suggests that DAPK1 expression is a potential prognostic biomarker in ccRCC patients.

### DAPK1 is a potential diagnostic biomarker in ccRCC patients

**Figure 3:**
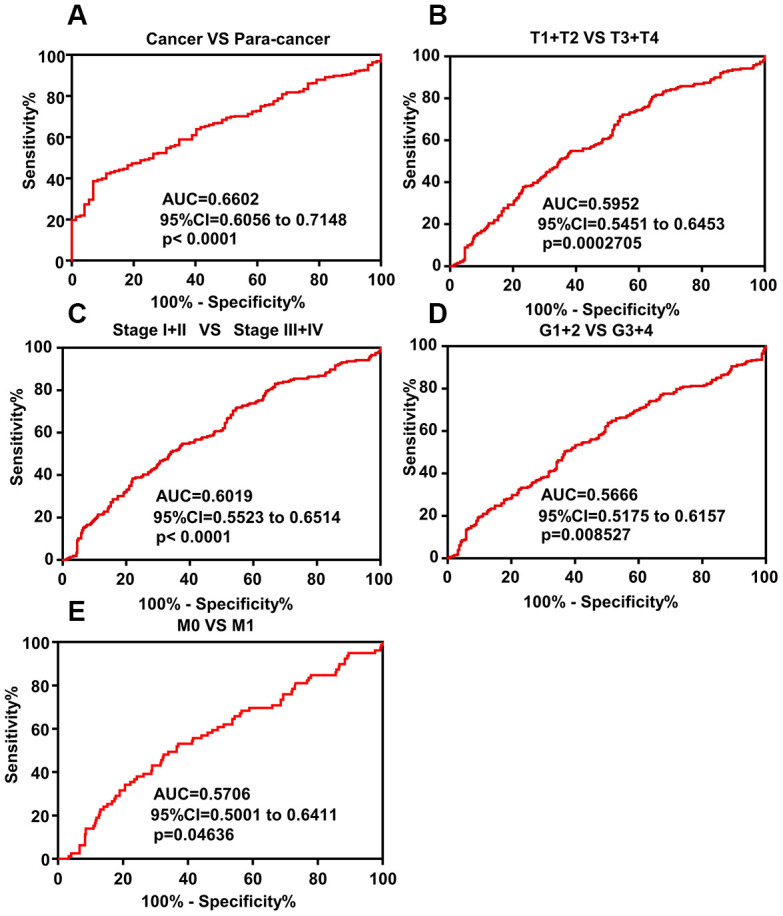
**ROC curve analysis shows diagnostic significance of DAPK1 expression in ccRCC patients.** (**A**) ROC curve analysis shows that DAPK1 expression accurately distinguishes ccRCC tissues from the corresponding normal kidney tissues with an AUC value of 0.6602 (95% CI: 0.6056 to 0.7148; p<0.0001). (**B**–**E**) ROC curve analysis shows that DAPK1 expression distinguishes advanced- and early-stage ccRCC patients based on (**B**) T stages (T1+T2 vs. T3+T4), (**C**) pathologic Stages (I+II vs. III+IV), (**D**) G stages (G1+G2 vs. G3+G4), and (**E**) M stages (M0 vs. M1).

ROC curve analysis was performed to evaluate the diagnostic significance of DAPK1 in ccRCC patients and showed that DAPK1 mRNA levels accurately distinguish ccRCC tissues from adjacent normal kidney tissue samples (AUC=0.6602; 95% CI: 0.6056-0.7148; p < 0.0001; Figure 3A). Moreover, ROC curve analysis of various subgroups of ccRCC patients showed that DAPK1 expression accurately distinguished early and advanced stage patients, including (T1 + T2)/(T3 + T4) stage (Figure 3B, AUC =0.5952, P<0.001), TNM (I + II)/(III + IV) stage (Figure 3C, AUC =0.6019, P<0.0001), (G1+G2)/(G3 + G4) stage (Figure 3D, AUC =0.5666, P=0.008527), M0/M1 (Figure 3E, AUC =0.5706, P=0.04636). This demonstrates that DAPK1 expression is a potential diagnostic biomarker for ccRCC.

### DAPK1 is an independent prognostic biomarker for predicting DFS in ccRCC patients

As shown in Table 2, multivariate analysis shows that N stage (HR, 3.427; 95% CI, 1.658-7.080; p = 0.001), M stage (HR, 3.940; 95% CI, 2.589-5.996; p <0.001), G grade (HR, 2.395; 95% CI, 1.565-3.666; p <0.001), pathological stage (HR, 3.003; 95% CI, 1.880-4.799; p <0.001) and DAPK1 expression (HR, 0.615; 95% CI, 0.422-0.896; p = 0.011) are independent prognostic factors that can predict DFS of ccRCC patients (Table 2). These results demonstrate that DAPK1 is an independent prognostic biomarker for predicting DFS in ccRCC patients.

**Table 2:** Univariate and multivariate analyses of clinicopathological factors that correlate with disease-free survival of ccRCC patients.

**Clinical variable**	**Univariate analysis**			**Multivariate analysis^c^**		
	**HR^a^**	**95% CI^b^**	**P-value**	**HR**	**95% CI**	**P-value**
**Disease Free survival (n=428)**						
Age (years)	1.377	0.967-1.963	0.076			
≤60 (n = 218)						
>60 (n = 210)						
Gender	1.389	0.939-2.056	0.100			
Male (n = 283)						
Female (n = 145)						
T stage	4.629	3.207-6.681	<0.001			
T1 or T2 (n = 286)						
T3 or T4 (n = 142)						
N stage	5.679	2.85-11.318	<0.001	3.427	1.658-7.080	0.001
N0 or NX (n = 415)						
N1 (n = 13)						
M stage	8.691	6.00-12.589	<0.001	3.940	2.589-5.996	<0.001
M0 or MX (n = 375)						
M1 (n = 53)						
G grade	3.481	2.306-5.253	<0.001	2.395	1.565-3.666	<0.001
G1 or G2 (n = 212)						
G3 or G4 (n = 216)						
Stage	6.671	4.474-9.946	<0.001	3.003	1.880-4.799	<0.001
I or II (n=273)						
III or IV (n=155)						
DAPK1	0.524	0.386-0.711	<0.001	0.615	0.422-0.896	0.011
Low(n = 214)						
High (n = 214)						

^a^ HR estimated from Cox proportional hazard regression model; ^b^ CI of the estimated HR; ^c^ Multivariate models were adjusted for T, N, M, and G grade classification and Stage; CI, confidence interval; HR, hazard ratio; DAPK1, death associated protein kinase 1.

### DAPK1 protein expression is downregulated in ccRCC patient tissues

**Figure 4:**
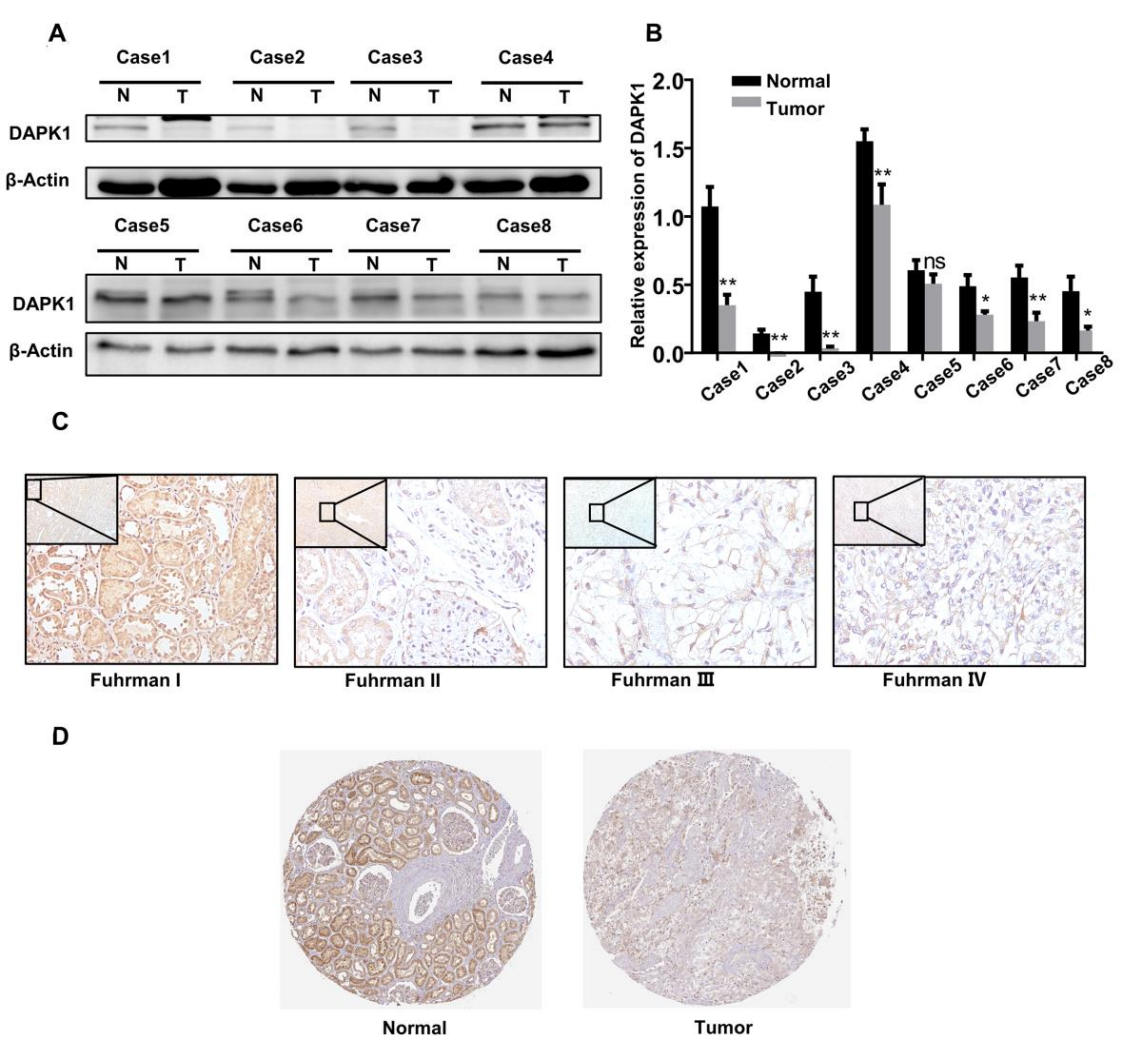
**DAPK1 protein expression is reduced in advanced ccRCC patient tissues.** (**A**, **B**) Western blotting and grayscale analysis results show DAPK1 protein expression in 8 pairs of ccRCC and adjacent normal kidney tissue samples. GAPDH is used as internal control. (**C**) Immunohistochemical (IHC) analysis shows DAPK1 protein expression in ccRCC tissues from patients with different Fuhrman stages. (**D**) IHC analysis shows DAPK1 protein expression in normal and ccRCC tissues based on IHC analysis in The Human Protein Atlas database.

Western blot and grayscale analysis of 8 paired ccRCC and adjacent normal kidney tissues showed that DAPK1 protein expression was significantly lower in ccRCC tissues compared to adjacent normal kidney tissues (Figure 4A–4B). Fuhrman grades is a recognized pathological grading system for renal cell carcinoma. The higher the grade, the higher the degree of malignancy. Immunohistochemical (IHC) analysis showed that higher Fuhrman grades were associated with decreased DAPK1 protein expression (Figure 4C). Moreover, analysis of the Human Protein Atlas (http://www.proteinatlas.org) database showed that DAPK1 protein expression was significantly lower in the advanced stages of ccRCC compared to the lower stages (Figure 4D).

### Biological function of DAPK1 in ccRCC

**Figure 5:**
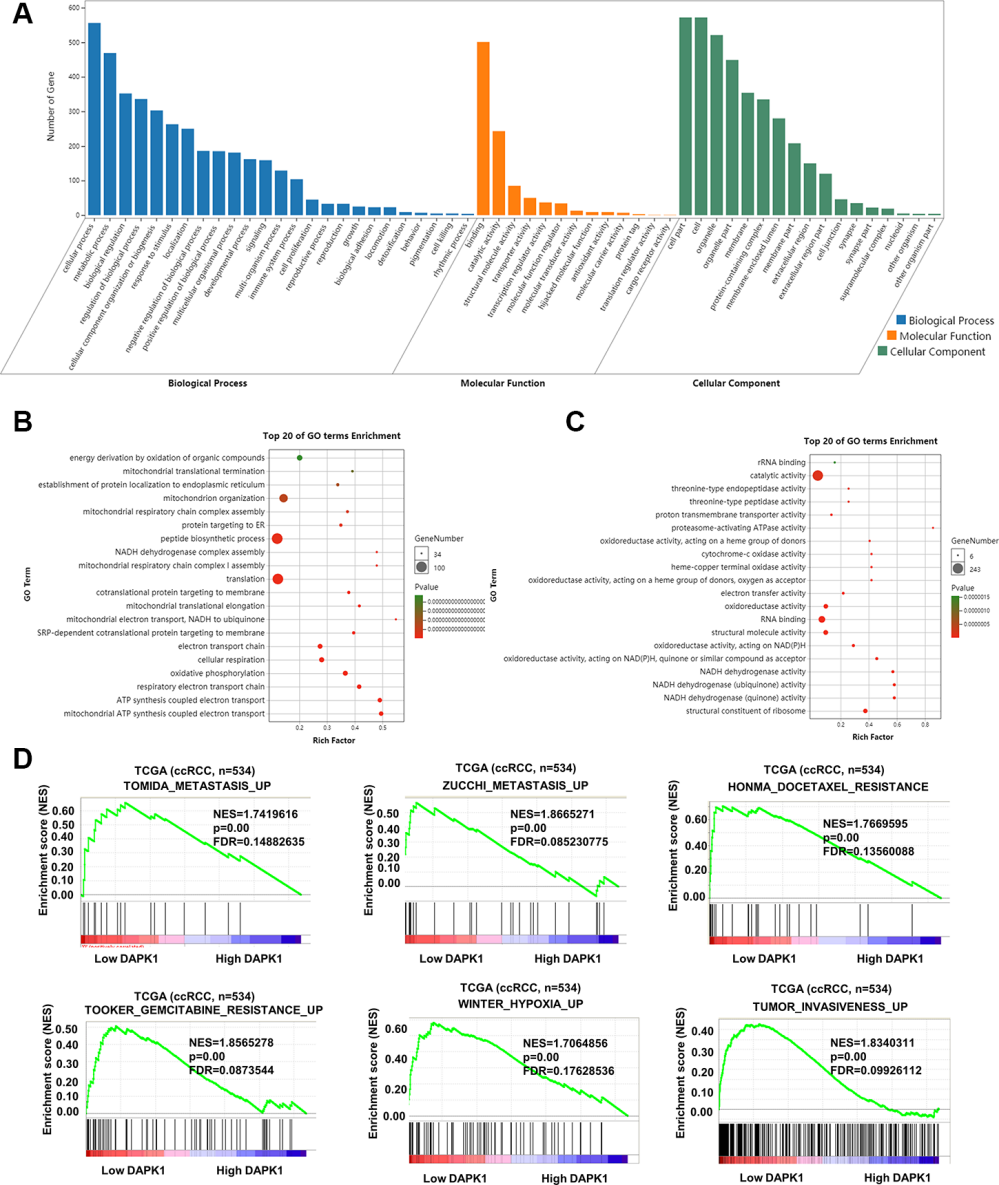
**Functional enrichment analysis of genes related to DAPK1 in ccRCC tissues.** (**A**–**C**) GO enrichment analysis shows the biological processes, molecular functions and cellular components that represent genes that negatively correlate with DAPK1 expression based on the transcriptome analysis of the TCGA-KIRC dataset. (**D**) GSEA show that genes upregulated because of DAPK1 downregulation in ccRCC include those that promote metastasis, drug resistance, hypoxia, and invasiveness. Note: ***p<0.001, **p<0.01, *p<0.05.

Next, we analyzed the ccRCC transcriptome data from the TCGA database to determine the biological function of DAPK1. GO analysis of genes that negatively correlate with DAPK1 expression (r < -0.3) showed that these genes were associated with signal pathways related to cellular growth, cell proliferation, endoplasmic reticulum, mitochondrion and others (Figure 5A–5C). Gene set enrichment analysis (GSEA) showed that low DAPK1 expression was associated with upregulation of pathways related to metastasis, drug resistance, hypoxia and invasiveness (Figure 5D). Protein-protein interaction (PPI) network analysis showed that DAPK1 was associated with apoptosis and negative feedback regulation of the MAPK pathway (Supplementary Figure 1B).

### DAPK1 overexpression promotes sunitinib sensitivity in sunitinib-resistant ccRCC cell lines

**Figure 6:**
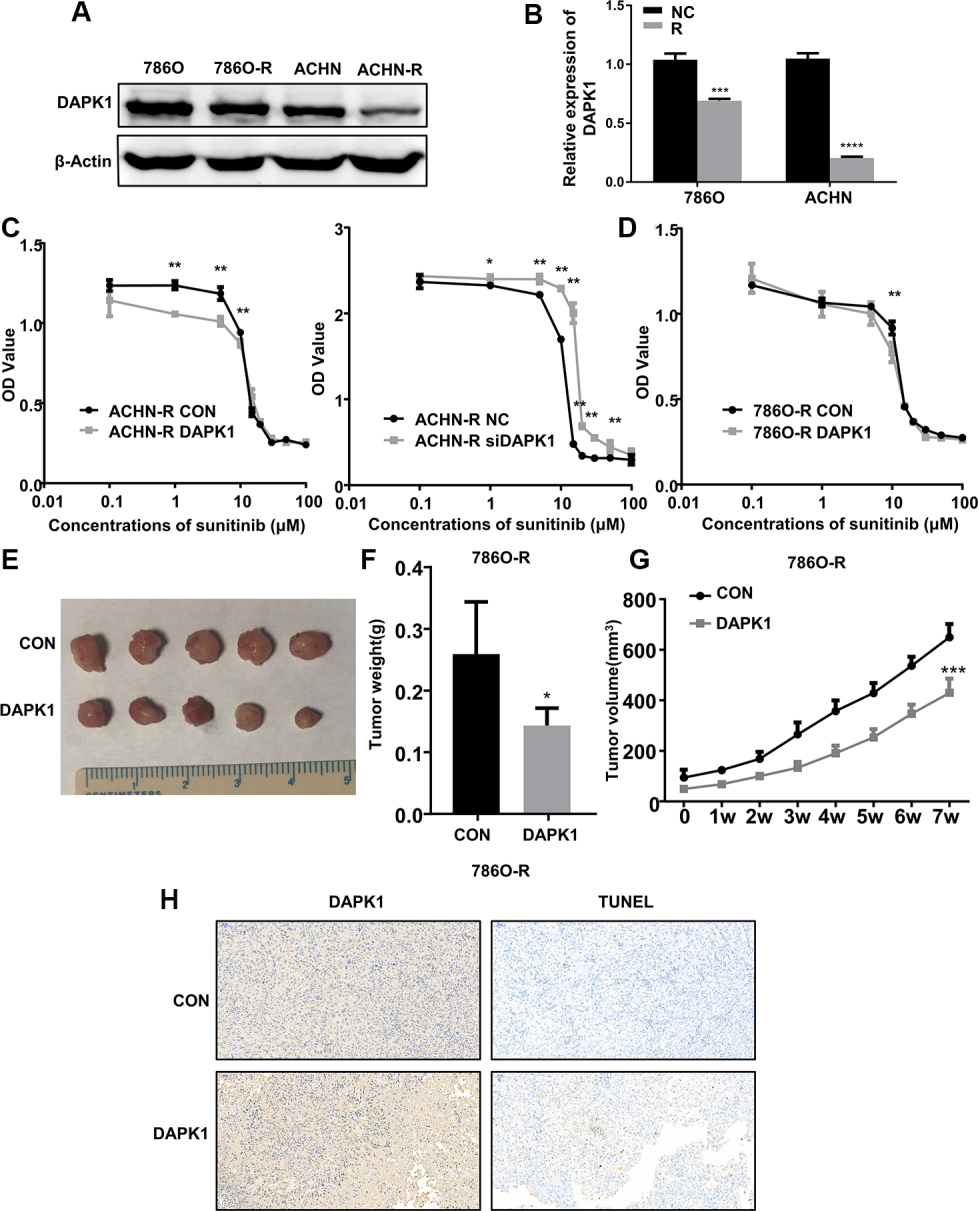
**DAPK1 overexpression in the sunitinib-resistant ccRCC cells reduces the growth of xenograft tumors in the nude mice model.** (**A**, **B**) Western blot analysis shows DAPK1 protein expression in parental (786-O and ACHN) and sunitinib-resistant (786O-R and ACHN-R) ccRCC cells. GAPDH is used as internal control. (**C**) CCK-8 assay results show the growth rate of control-ACHN-R, DAPK1-overexpressing ACHN-R, and DAPK1-knockdown ACHN-R cells that are treated with different concentrations of sunitinib. (**D**) CCK-8 assay results show the growth rate of control and DAPK1 overexpressing 786O-R cells that are treated with different concentrations of sunitinib. The picture (**E**), average weight (**F**) and volume (**G**) of the tumors obtained after 7 weeks of transplanting control and DAPK1 overexpressing 786O-R cells in the nude mice. (**H**) Immunohistochemical analysis of the xenograft tumors obtained by subcutaneously transplanting control and DAPK1 overexpressing 786O-R cells in nude mice. The bar graphs show the statistical analysis of three independent experiments. Note: The data are shown as means ± SEM. ***p<0.001, ** p<0.01, *p<0.05.

Since we observed a close relationship between DAPK1 and metastasis as well as drug resistance in ccRCC patients, we analyzed DAPK1 expression in two sunitinib-resistant cell lines, 786-O-R and ACHN-R [16] by western blotting and immunofluorescence assays. The results showed that DAPK1 protein levels were significantly lower in the 786-O-R and ACHN-R cells compared to their corresponding parental cell lines, 786-O and ACHN, respectively (Figure 6A–6B; Supplementary Figure 1C).

Previous studies demonstrate that DAPK1 decreases tumor cell proliferation and metastasis by promoting apoptosis in several cancers, including colorectal cancer, breast cancer, gastric cancer [15, 17–21]. Moreover, our results show that low DAPK1 levels correlates with poor prognosis and sunitinib resistance in ccRCC patients. Therefore, we tested if DAPK1 overexpression can overcome the sunitinib resistance of 786-O-R and ACHN-R cells. CCK-8 assay results show that DAPK1 overexpression significantly increases sunitinib sensitivity of 786O-R and ACHN-R cells, whereas, DAPK1 knockdown significantly increased sunitinib resistance of ACHN-R cells (Figure 6C, 6D). These results demonstrate that DAPK1 overexpression promotes sunitinib sensitivity in ccRCC cells.

### DAPK1 overexpression reduces the *in vivo* growth of sunitinib-resistant ccRCC cells in the nude mice model

Next, we subcutaneously injected control or DAPK1-overexpressing 786-O-R cells into nude mice to study the *in vivo* effects of DAPK1 overexpression on the growth of sunitinib-resistant ccRCC cells. After 4 weeks, the volume and weight of tumors obtained from DAPK1-overexpressing 786O-R cells was significantly reduced compared to those derived from the control 786O-R cells (Figure 6E–6G). Immunohistochemical analysis of xenograft tumor sections showed significantly higher levels of apoptosis in the xenograft tumors derived from DAPK1 overexpressing 786O-R cells compared to the corresponding controls (Figure 6H).

### DAPK1 triggers ER stress-induced apoptosis

**Figure 7:**
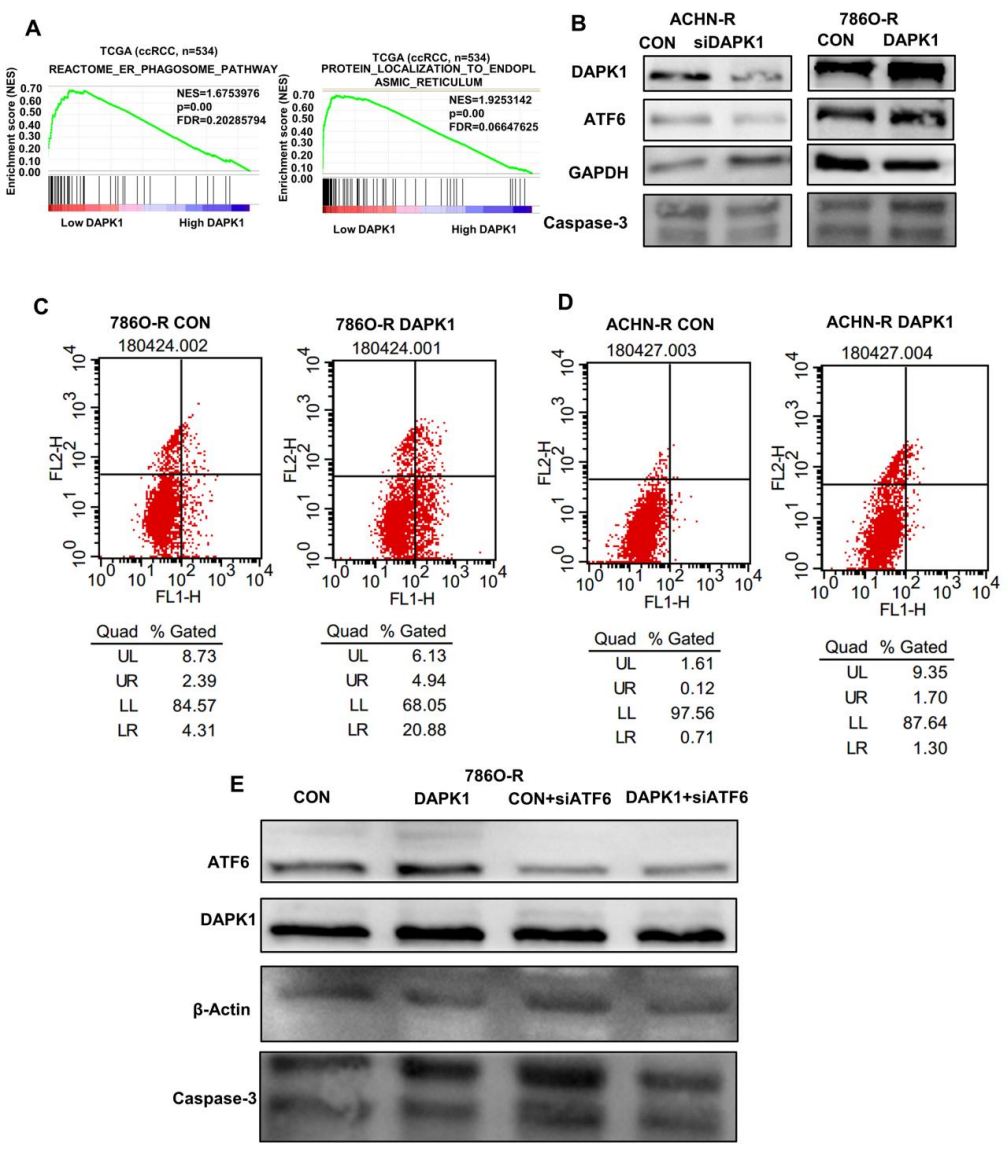
**DAPK1 regulates endoplasmic reticulum stress-mediated apoptosis.** (**A**) GSEA shows that DAPK1 expression correlates with reactome_er_phagosome_pathway and protein_localization_to_endoplasmic_reticulum pathway. (**B**) Western blotting shows ATF6 protein levels in control and DAPK1 knockdown ACHN-R, and control and DAPK1-overexpressing 786O-R cells. GAPDH is used as internal control. (**C**, **D**) Flow cytometry analysis shows the percentage of apoptotic cells in control and DAPK1 overexpressing 786O-R and ACHN-R cells. (**E**) Recovery experiment to verify the regulation of ATF6 by DAPK1.

Next, we analyzed if DAPK1 regulates apoptosis induced by ER stress in TCGA-KIRC dataset. GSEA results demonstrate that DAPK1 is associated with signaling pathways related to ER, including reactome_er_phagosome_pathway and protein_localization_to_endoplasmic_reticulum (Figure 7A). Western blotting analysis shows that DAPK1 overexpression in 786O-R cells upregulates levels of a ER stress-related protein, ATF6 (Figure 7B). Moreover, DAPK1 overexpression increases apoptosis in sunitinib-resistant 786O-R and ACHN-R cells compared to the corresponding controls (Figure 7C, 7D). Furthermore, recovery experiments show that after overexpression of DAPK1, the expression of Caspase-3 in 786O-R cell line increased significantly, while knocking down ATF6, on the basis of overexpression DAPK1, did not increase the expression of Caspase-3. This result showed that DAPK1 overexpression promoted apoptosis of sunitinib-resistant cells via upregulation of ATF6 (Figure 7E).

## DISCUSSION

Our study demonstrates that DAPK1 is an independent prognostic factor in ccRCC patients and its expression correlates with tumor stages and grades. Low DAPK1 expression correlates with the upregulation of genes involved in metastasis, drug resistance, tumor invasiveness, and the ER phagosome pathway. Moreover, DAPK1 expression is reduced in sunitinib-resistant ccRCC cell lines. DAPK1 overexpression induces apoptosis in sunitinib-resistant ccRCC cell lines by enhancing ATF6-dependent ER stress pathway.

DAPK1 is a pro-apoptotic serine-threonine protein kinase that is downregulated in lung, colon, breast, and liver cancers [15, 17–20]. In breast cancer patients, downregulation of DAPK1 expression is associated with tumor metastasis and recurrence [21]. The loss of DAPK1 expression is associated with poor prognosis and advanced tumor stages in lung cancer patients [22–24]. Moreover, hypermethylation of the DAPK1 promoter is reported in tumor tissues and lymph nodes of patients with gastric cancer and head and neck tumors [25–27]. DAPK1 promoter hypermethylation correlates with reduced sensitivity to radiochemotherapy in stage I non-small-cell lung cancer patients [28]. These studies showed that DAPK1 promoter hypermethylation was positively associated with advanced cancer stages and metastasis in several cancers, thereby suggesting that DAPK1 methylation is a potential prognostic marker in different human cancers. In ccRCC, inhibition of miR-34a-5p upregulates DAPK1 protein expression and corrects the dysregulated p53-DAPK axis [29].

Non-specific receptor tyrosine kinase inhibitors (RTKIs) such as sorafenib and sunitinib are the first-line therapy for patients with progressive and metastatic renal cancer [30]. However, majority of ccRCC patients develop sunitinib-resistance and hence several investigations are underway to determine the mechanisms that result in sunitinib resistance. Our study demonstrates that DAPK1 is downregulated in sunitinib-resistant ccRCC cell lines and its overexpression increases sensitivity to sunitinib by promoting apoptosis.

The endoplasmic reticulum (ER) is an important organelle for various cellular functions, including calcium homoeostasis, biosynthesis of sterols, lipids, and proteins, and post-translational modifications of newly synthesized proteins [31, 32]. Deregulation of redox or calcium homoeostasis, glucose deficiency or viral infections can induce ER stress-mediated unfolded protein response or UPR [33, 34]. If ER stress prolongs and is not resolved, it triggers apoptosis [35, 36]. UPR is mediated by several ER stress protein sensors such as ATF6, IRE1α, and PERK [31, 37]. A previous study reported that the levels of ER stress-related proteins are reduced in DAPK knock-out fibroblasts [13]. Moreover, in DAPK null fibroblasts, ER-stress induced caspase and autophagy activation was significantly reduced [13]. This suggests that DAPK is an important modulator of ER stress and regulates autophagy and caspase-induced apoptosis. Our study demonstrates that DAPK1 regulates ATF6 expression and promotes apoptosis in sunitinib-resistant cell lines. However, we did not observe significant changes in the levels of two other ER stress sensors, IRE1α, and PERK (data no shown). Our data therefore shows that DAPK1 is a potential therapeutic target to overcome sunitinib resistance in ccRCC patients.

In conclusion, we demonstrate the prognostic role and the mechanism of action of DAPK1 in ccRCC patients. We demonstrate that low DAPK1 expression is a potential prognostic biomarker for ccRCC patients. DAPK1 overexpression reverses sunitinib resistance in sunitinib-resistant ccRCC cell lines by inducing ATF6-related ER stress and apoptosis. Therefore, DAPK1 is a potential prognostic biomarker and therapeutic target in ccRCC patients, but, the results of our study need to be investigated further in larger prospective clinical trials.

## MATERIALS AND METHODS

### Patient tissues

We obtained 8 pairs of primary ccRCC and adjacent normal kidney specimens from the Department of Urology, The Central Hospital of Wuhan (Wuhan, China). The adjacent normal kidney tissue specimens were obtained 5 cm from the site of primary tumor. The tissues were immediately frozen in liquid nitrogen and stored at -80°C for protein extraction. We obtained informed consent from all patients. This study was authorized by the Human Research Ethics Committee of the Huazhong University of Science and Technology.

### Cell culture

The ACHN and 786-O ccRCC cell lines from The American Type Culture Collection (ATCC; Manassas, VA, USA). The ccRCC cells were grown in Dulbecco's modified Eagle's medium (DMEM; Google Biotechnology Co., Ltd, Wuhan, China) containing 10% FBS and 1% penicillin-streptomycin in a humidified incubator 37°C and 5% CO_2_ as previously described [38], In our previous study, we obtained sunitinib-resistant cell lines by continuously adding low-dose sunitinib to the cell culture medium and intermittently adding high-dose sunitinib, which were named 786O-R and ACHN-R [16, 38].

### Western blotting

RIPA lysis buffer was used to extract the total tissue and cellular proteins. The protein concentrations were determined using the BCA Protein Assay Kit (Thermo Scientific). Equal amounts of tissue or cellular protein extracts were separated on 10% or 12% SDS-PAGE gels and transferred onto PVDF membranes (Millipore, Eschborn, Germany). The membranes were blocked with 5% nonfat milk for 1-2 h at room temperature. Then, the blots were incubated overnight at 4 °C with primary antibodies against DAPK1 (25136-1-AP; ProteinTech), ATF6 (ab227830; Abcam), GAPDH (ab198394; Abcam), and Caspase-3 (#9662; Cell Signaling Technology). The blots were then probed with the corresponding HRP-conjugated secondary antibodies (ab150117, ab150079; Abcam) for 1-2 h at room temperature. The blots were developed using Enhanced Chemiluminescence kit (ECL; Bio-Rad Laboratories, Inc., Hercules, CA, United States). The amounts of DAPK1, ATF6 and Caspase-3 were quantified relative to GAPDH using the ChemiDoc-XRsþ (Bio-Rad).

### Transient transfection assays

We obtained the DAPK1 expression plasmid (DAPK1) and the corresponding control vector (SLPI pENTER vector cloning) from Vigene Biosciences (Shandong, China) and transfected ccRCC cell lines with 2 μg DAPK1 or the corresponding vector using lipofectamine 2000 according to the manufacturer's recommendations. The siRNA oligonucleotide sequences aiming at DAPK1 (siDAPK1) and the siRNA negative control (si-NC) were purchased by GenePharma (Shanghai, China). Based on the manufacturer's protocols, si-DAPK1 and si-NC with a final concentration of 50 nM were mixed with Lipofectamine® 2000 (Invitrogen, USA). The DAPK1 sequences were as follows: #1: 5′- CCACGTCGATACCTTGAAATT-3′;

### Immunohistochemistry and Immunofluorescence assay

Mainly, the tissue specimens were fixed with formalin, dehydrated and embedded in paraffin. The tissue sections were incubated with rabbit DAPK1 polyclonal antibody (1:400 ProteinTech) overnight at 4 °C. The next day, the tissue sections were washed with PBS and then incubated with secondary antibodies. We performed immunofluorescence assays with DAPK1 primary antibody at 4 °C for 6-8 h. Next, the cells were blocked with CY3-conjugated secondary antibodies for 1 hour and DAPI was added as previously described [36].

### Cell apoptosis assay

We measured cellular apoptosis using the Apoptosis Detection Kit according to the manufacturer’s instructions. We first collected the DAPK1- or vector control-transfected 786O-R and ACHN-R cells using 0.125% trypsin. Then, the cells were rinsed with PBS and resuspended in Annexin V binding buffer containing 5ul FITC-conjugated Annexin V for 15-20 min at 4 °C in the dark. Then, after washing with PBS buffer, the cells were stained with 5ul propidium iodide (PI) and immediately analyzed using the MACSQuant FACS Analyzer (Teterow, Germany). Then, the percentage of apoptotic cells in each sample, that is, Annexin-V-FITC^+^ PI^+^ and Annexin-V-FITC^+^ PI^-^ were determined using the FlowJov7.6 software.

### CCK-8 assay

We analyzed the *in vitro* growth of ccRCC cells using the CCK8 assay kit (Dojindo, Kumamoto, Japan) according to the manufacturer's instructions. We seeded 2 × 10^4^ cells per 100 μl medium in 96-well plates and added different concentrations (0.1, 1, 5, 10, 15, 20, 30, 50, 100uM) of sunitinib or placebo to their corresponding wells. After 48 h, the medium was replaced by 100 μl serum-free medium with 10 μl CCK8 solution to each well and incubated at 37 °C for another 2 h. Then, the absorbance of each well corresponding to the cell viability was determined at 450 nm in a plate reader.

### Bioinformatics analysis

We obtained the DAPK1 mRNA expression and the clinicopathological features of 533 ccRCC patients tumor tissues and 72 Adjacent normal tissues from the cBioPortal (http://www.cbioportal.org/public-porta). We also obtained DAPK1 gene expression for ccRCC patients from the Oncomine database (https://www.oncomine.org). The expression of DAPK1 protein in RCC tissues based on immunohistochemistry (IHC) was obtained from The Human Protein Atlas (http://www.proteinatlas.org) database. We performed Gene set enrichment analysis (http://www.gsea-msigdb.org/gsea/index.jsp) to analyze the signaling pathways related to DAPK1. We also performed protein-protein interaction (PPI) network analysis used The Search Tool for the Retrieval of Interacting Genes (STRING) and Implemented Gene ontology (GO) analysis as previously described [39].

### Xenograft tumors in nude mice

We purchased 4- to 5-week-old male nude mice from Beijing HFK Company (Beijing, China) and injected 5×10^6^ in 100 μl of control or DAPK1-transfected 786O-R cells into the right armpit. The tumor volumes (V) were documented once a week for 7 weeks. The mice were euthanized after 7 weeks and the tumors were isolated and stored at -80°C for further experiments. The volume of the tumor was calculated according to the length (L) and width (W). The calculation formula was V= (L×W^2^)/2. All animal experiments were conducted as approved by the Ethics Committee of Tongji Medical College, Huazhong University of Science and Technology.

### Statistical analysis

All statistical analysis was performed using the SPSS Statistics 22.0 software (IBM SPSS, Chicago, USA). Statistical differences between experimental groups were analyzed using the Student’s t-test. The Kaplan–Meier (KM) curve analysis was used to evaluate the relationship between DAPK1 expression and survival rates. Receiver operator characteristic (ROC) curve analysis was performed and area under the curve (AUC) values was calculated to determine the prognostic accuracy of DAPK1 expression as a parameter. The univariate and multivariate Cox proportional hazard regression analyses were performed to assess the prognostic significance of DAPK1 in RCC. Two-tailed P < 0.05 was considered statistically significant.

## Supplementary Materials

Supplementary Figure 1
