# Supplementary material for: Low DAPK1 expression correlates with poor prognosis and sunitinib resistance in clear cell renal cell carcinoma
Source: Aging (Albany NY). 2020 Nov 16;13(2):1842–58. doi: 10.18632/aging.103638 (PMC7880360; doi:10.18632/aging.103638)
Supplement: Supplementary Figure 1 [file aging-13-103638-s001.pdf]

SUPPLEMENTARY FIGURE

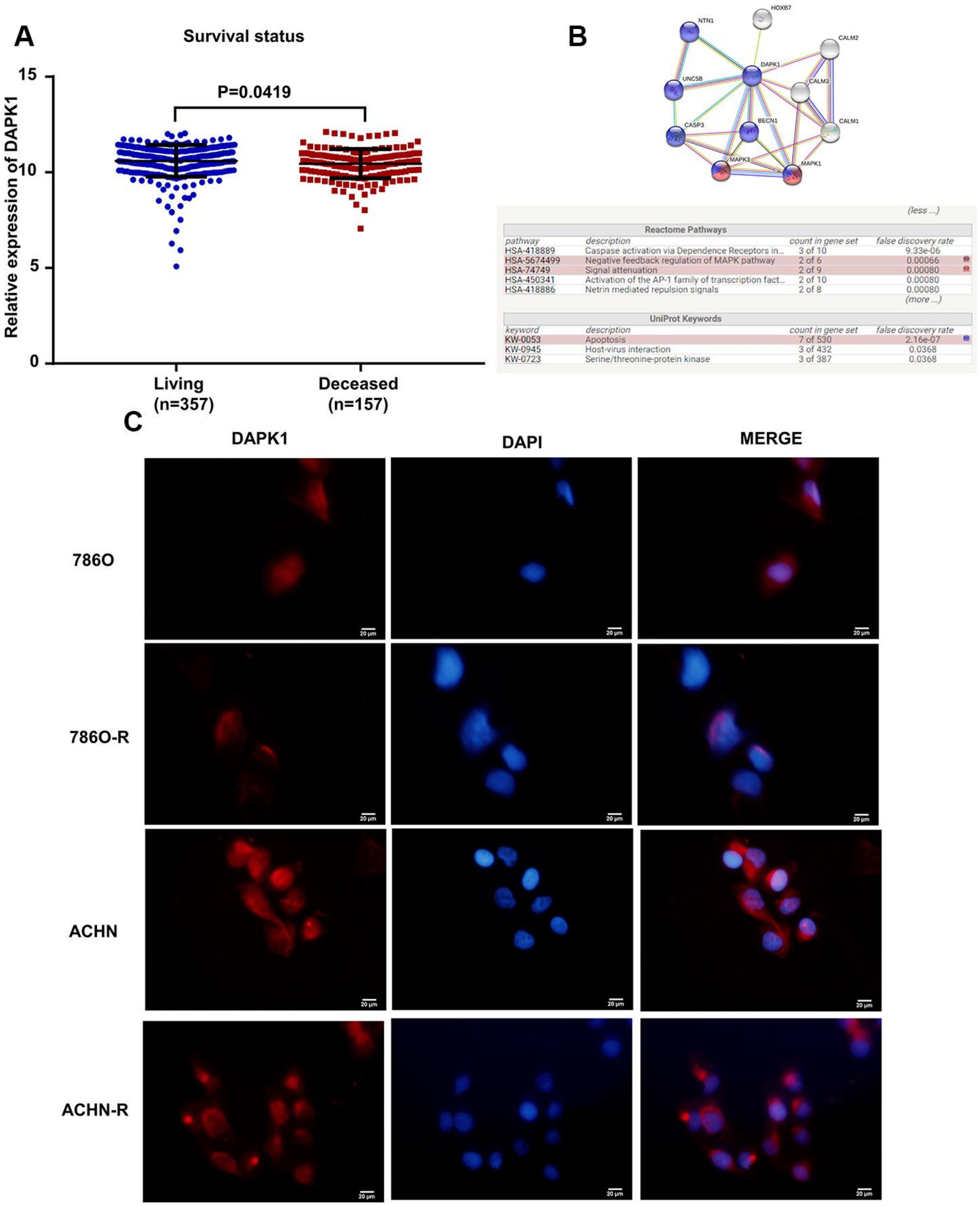

Supplementary Figure 1. PPI network analysis of DAPK1-interacting genes and DAPK1 expression in sunitinib-resistant ccRCC cells. (**A**) In TCGA-KIRC dataset, the DAPK1 mRNA levels in ccRCC patients with distinct survival status. (**B**) PPI network analysis shows the genes that interact with DAPK1. (**C**) Immunofluorescence assay shows DAPK1 expression in parental cells and sunitinib-resistant ccRCC cells. The bar graphs show the means ± SEM of three independent experiments. ***p<0.001, **p<0.01, *p<0.05.
